# Cognitive Performance During Confinement and Sleep Restriction in NASA’s Human Exploration Research Analog (HERA)

**DOI:** 10.3389/fphys.2020.00394

**Published:** 2020-04-28

**Authors:** Jad Nasrini, Emanuel Hermosillo, David F. Dinges, Tyler M. Moore, Ruben C. Gur, Mathias Basner

**Affiliations:** ^1^Unit for Experimental Psychiatry, Department of Psychiatry, University of Pennsylvania Perelman School of Medicine, Philadelphia, PA, United States; ^2^Brain Behavior Laboratory, Department of Psychiatry, University of Pennsylvania Perelman School of Medicine, Philadelphia, PA, United States

**Keywords:** cognition, performance, spaceflight, spaceflight analog, NASA, sleep deprivation, confinement

## Abstract

Maintaining optimal cognitive performance in astronauts during spaceflight is critical to crewmember safety and mission success. To investigate the combined effects of confinement, isolation, and sleep deprivation on cognitive performance during spaceflight, we administered the computerized neurobehavioral test battery “Cognition” to crew members of simulated spaceflight missions as part of NASA’s ground-based Human Exploration Research Analog project. Cognition was administered to *N* = 32 astronaut-like subjects in four 1-week missions (campaign 1) and four 2 weeks missions (campaign 2), with four crewmembers per mission. In both campaigns, subjects performed significantly faster on Cognition tasks across time in mission without sacrificing accuracy, which is indicative of a learning effect. On an alertness and affect survey, subjects self-reported significant improvement in several affective domains with time in mission. During the sleep restriction challenge, subjects in campaign 1 were significantly less accurate on a facial emotion identification task during a night of partial sleep restriction. Subjects in campaign 2 were significantly slower and less accurate on psychomotor vigilance, and slower on cognitive throughput and motor praxis tasks during a night of total sleep deprivation. On the survey, subjects reported significantly worsening mood during the sleep loss challenge on several affective domains. These findings suggest that confinement and relative isolation of up to 2 weeks in this environment do not induce a significant negative impact on cognitive performance in any of the domains examined by Cognition, although the concurrent practice effect may have masked some of the mission’s effects. Conversely, a night of total sleep deprivation significantly decreased psychomotor vigilance and cognitive throughput performance in astronaut-like subjects. This underscores the importance of using cognitive tests designed specifically for the astronaut population, and that survey a range of cognitive domains to detect the differential effects of the wide range of stressors common to the spaceflight environment.

## Introduction

Space exploration missions will require astronauts to adapt to life-threatening environments for significantly longer periods of time than current low earth orbit missions. The success of exploration missions will depend critically on astronauts’ intact neurobehavioral functions. Yet the spaceflight environment consists of many risk factors that can have a negative impact on cognition [e.g., microgravity, hypercapnia (Manzey and Lorenz, 1998), hypoxia (Lieberman et al., 2005; Elmenhorst et al., 2009), and radiation (Hienz et al., 2008; Manda et al., 2008; Liu et al., 2010)]. Other stressors such as high workload, sleep restriction, circadian misalignment, confinement, and isolation can also pose threats to the ability of astronauts to sustain high levels of cognitive performance over prolonged periods of time. Astronauts have described neurobehavioral deficits during spaceflight, including befuddlement, altered time sense, dizziness, slowing, poor concentration, and the need to perform tasks very slowly and precisely, especially after initial exposure to the spaceflight environment (Hickie et al., 1997; Kanas et al., 2001; Welch et al., 2009; Sandoval et al., 2010). It is unclear to what extent discrepancies between astronauts’ subjective reports and their objective cognitive performance can be explained by small sample sizes, the lack of control groups, practice effect confounds, and/or the use of neurobehavioral tests that have not been designed for highly motivated, high performing astronaut populations, and may thus not have been sensitive enough (Strangman et al., 2014). This highlights the critical need for comprehensive, sensitive and validated neurobehavioral assessments in spaceflight in general, and during exploration type missions specifically, to aid mission success.

This study systematically assessed cognitive performance in crewmembers of two campaigns at NASA’s ground-based Human Exploration Research Analog (HERA) project. Volunteer crewmembers were confined to the HERA ground-based facility at Johnson Space Center across eight missions in two campaigns for one or two weeks, respectively, and performance was assessed using the Cognition test battery (Basner et al., 2015). The battery consists of 10 validated and brief neurobehavioral tests that cover an array of cognitive domains and that were specifically developed for high-performing astronauts by our research team (see methods).

## Methods

### The HERA Facility

The HERA facility is a two-story habitat at NASA’s Johnson Space Center. It is cylindrical with a vertical axis divided into a core chamber and a loft, and connects to a simulated airlock and hygiene module. The total habitable volume of the facility is 148.6 m^3^, divided into four distinct sections: the core (56.0 m^3^), loft (69.9 m^3^), airlock (8.6 m^3^), and hygiene module (14.1 m^3^) (NASA, 2019). The HERA project represents an analog to simulate the effects of isolation, confinement, and the remote conditions of exploration mission scenarios. Studies in HERA simulations typically include, but are not limited to, behavioral health and performance assessments, communication and autonomy studies, human factors evaluations, and exploration medical capabilities assessments and operations. For a schematic diagram of the HERA facility see NASA’s HERA facility and capabilities information document (2019).

### HERA Campaign and Simulation Structure

A HERA campaign was defined as one integrated protocol with a single primary mission scenario and consisted of four missions in order to meet study subject number requirements.

HERA campaign 1 consisted of four 1-week periods of confinement and started in February 2014 with mission 1 (C1M1) and concluded in September 2014 with mission 4 (C1M4). HERA campaign 2 consisted of four 2-week periods of confinement and started in January 2015 with mission 1 (C2M1) and concluded in September 2015 with mission 4 (C2M4). Each mission was preceded by a 2-week period designated for baseline data collection and another 1- or 2-week period for post-mission data collection.

Mission days in HERA were operationally structured comparably to current operations on the International Space Station (ISS). They included research tasks, operational tasks, emergency simulations, and a stress induction test (in C2 only). Mission control monitored activities in the facility 24/7. They could see and hear via audiovisual feeds what was going on in the facility at all times except during periods where communication time delays were simulated. Communication of researchers with the crew was relayed through mission control.

**FIGURE 1:**
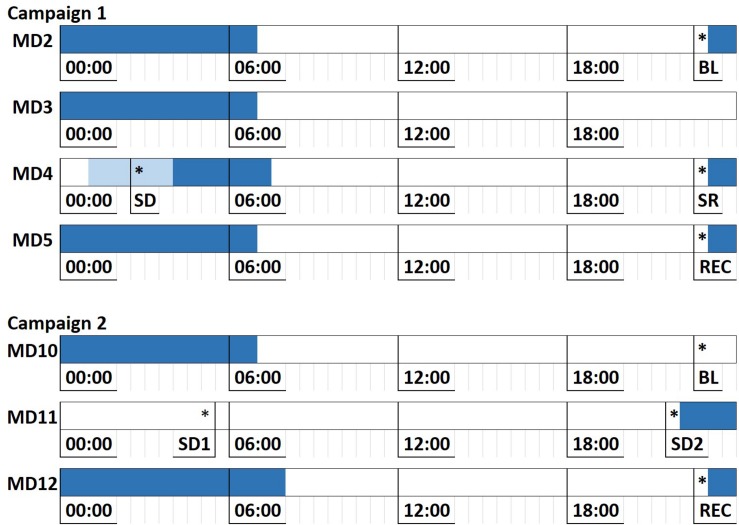
Sleep deprivation/restriction implementation across HERA campaigns 1 and 2. Each line represents a full mission day, with mission day number denoted to the left. Dark blue areas denote scheduled sleep opportunity. Light blue area in C1 MD4 denotes the range of sleep opportunity times due to variation in sleep schedule across missions in campaign 1. Asterisks denote average time of administration of the Cognition test battery across missions. MD, Mission Day; BL, Baseline; SD (1/2), Sleep Deprivation; SR, Sleep Restriction; REC, Recovery.

Bed time was typically scheduled from 11 p.m. until 7 a.m., with one night of sleep restriction or deprivation in each campaign: In campaign 1, subjects were only allowed to go to bed around 3 am on the night of Mission Day 3 (MD3), with some variations across missions. In campaign 2, subjects were sleep deprived for one full night (from MD10 to MD11), then given a slightly increased sleep opportunity of 10 h on MD11 after being awake for ≈40 h (Figure 1).

The mission simulations in HERA maintain high fidelity to many operational features of actual spaceflight. Crew size is limited to 4 subjects per study, for instance, to simulate the small crew sizes of anticipated exploration spaceflight missions. Similarly, communication to the outside world is restricted through a simulated mission control center, with a weekly scheduled family conference, which simulates the relative isolation of typical spaceflight missions. The physical features of the HERA habitat itself mimic the confinement of a spacecraft environment. Other logistic features of the missions like type and duration of activities, diet, access to exercise, and the introduction of sleep challenges and stressful tasks are all designed to replicate the operational challenges of spaceflight (NASA, 2019). Conversely, other spaceflight stressors that may impact cognitive performance are not represented in HERA, like microgravity, perceived threat to life, chronically elevated levels of CO_2_, significantly longer mission durations, and (in the case of travel beyond low earth orbit) radiation. As such, the effects of the simulated missions in the HERA environment are best taken in combination with studies in other environments to fully understand the impact of various spaceflight stressors on cognitive function. In spite of that, the mission simulations at HERA offer some of the highest fidelity reproductions possible of the spaceflight logistic environment due to their functional proximity to NASA’s actual spaceflight operations procedures.

### Subjects

Each mission consisted of a crew of four. Participants were volunteers selected by NASA’s Flight Analogs Project. All subjects were required to pass a screening for the federally regulated Third-Class Airman Medical Certificate (2019), as well as psychological screening administered by NASA’s Behavioral Health and Performance Operations, to be eligible for participation. Subject selection prioritized individuals considered to be “astronaut-like” in terms of age range, technical skill, and educational attainment or equivalent military experience (NASA, 2019). Subjects gave written informed consent prior to participation. They were free to discontinue the study at any time. The study was approved by NASA’s Institutional Review Board.

A total of 32 unique subjects (mean age 36.0 years, SD 7.6 years, range 27–53 years, 17 female) participated in the 8 HERA missions in groups of four. Subjects were not permitted to participate in multiple missions. 29 (90%) crewmembers were right-handed. In terms of their highest level of educational attainment, 6 (19%) had a Bachelor’s degree, 15 (47%) had a Master’s degree, and 11 (34%) had a Ph.D. or equivalent.

Except for C1M2 with an all-female crew, and C2M1with three male crew members, crews were gender balanced with 2 male and 2 female participants. Crewmembers were each assigned one of the following mission roles before the start of a mission: Commander (CDR), Flight Engineer (FE), Mission Specialist 1 (MS1), or Mission Specialist 2 (MS2).

### The Cognition Test Battery

The Cognition test battery is a computerized neurobehavioral test battery designed specifically for the high-performing astronaut population (Basner et al., 2015). Cognition consists of 15 unique versions of 10 neurobehavioral tests that cover a range of cognitive domains. These unique versions systematically vary the stimuli within each task to allow repeated administration of the battery while minimizing ceiling effects in learning due to memorizing the stimulus set. Table 1 shows the 10 individual tests within the battery and the cognitive domains assessed by each. The Cognition test battery is described in detail in Basner et al. (2015). In HERA, Cognition was administered on Apple iPads (4th generation) with the Joggle Research software (Pulsar Informatics Inc., Philadelphia, PA, United States). Each crewmember had a personal iPad dedicated for Cognition administration. The iPad iteration of Cognition has been validated by Moore et al. (2017) in a sample of highly educated adults, but some overall differences between the iPad and Laptop iterations of the battery were found, especially on overall speed. While these differences do not impact comparisons within subjects on a single platform, they do render direct comparisons between the laptop and iPad platforms problematic.

**TABLE 1:** The 10 tasks of the cognition test battery and the cognitive domains assessed by each.

**Task abbreviation**	**Full name**	**Cognitive domain assessed**
MP	Motor praxis	Sensory-motor speed
VOLT	Visual object learning task	Spatial learning and memory
F2B	Fractal 2-back	Working memory
AM	Abstract matching	Abstraction, concept formation
LOT	Line orientation task	Spatial orientation
ERT	Emotion recognition task	Emotion identification
MRT	Matrix reasoning task	Abstract reasoning
DSST	Digit symbol substitution test	Complex scanning and visual tracking, working memory
BART	Balloon analog risk task	Risk decision making
PVT	Psychomotor vigilance test	Vigilant attention

HERA crewmembers watched a standardized familiarization video that explained software handling and each of the 10 Cognition tests before they performed Cognition for the first time for familiarization. During this familiarization session, they were required to complete a practice version of each test before taking the actual test (available for 8 out of the 10 Cognition tests). For subsequent administrations, taking the practice version of a test was optional. Crewmembers completed the full test battery daily throughout each mission, as well as before and after the mission, according to the schedule below:

• 3 times pre-mission
  ○ Campaign 1: MD-10, MD-3, MD-1
  ○ Campaign 2: MD-8/9, MD-4, MD-1
• On a daily basis during missions
  ○ Campaign 1: MD1 – MD7
  ○ Campaign 2: MD1 – MD14
• Once additionally in each mission of campaign 2 during the sleep deprivation night.
• 3 times post mission
  ○ Campaign 1: MD + 1, MD + 6/7, MD + 14
  ○ Campaign 2: MD + 1, MD + 5, MD + 7

During each mission, Cognition administration was scheduled immediately prior to scheduled bed time (with some exceptions during sleep deprivation periods; see Figure 1). During pre- and post-mission testing, administration time was flexible but restricted to prevent testing within a period of 1 h after waking up (to avoid sleep inertia) or after a cumulative time of 16 h awake (to avoid sleep deprivation). Subjects were instructed to take the test as a group at the same time, and to avoid distracting the other crewmembers if finished early.

The 15 unique versions of Cognition were always administered in the same order starting with battery #1 (i.e., #1, #2, #3, etc.) to facilitate comparisons to normative data gathered in astronauts, astronaut candidates and mission controllers (Basner et al., 2015). In campaign 2, the battery sequence started again with battery #1 after battery #15, as Cognition was administered more than 15 times total.

### Surveys

A survey was administered in each HERA campaign immediately prior to each administration of the Cognition battery, but different surveys were used for each campaign. The survey administered in campaign 1 (ISS Survey) was a brief alertness survey conducted using paper and pencil, contains 4 questions, and is identical to the survey currently administered on the ISS and in the ground study in astronauts, astronaut candidates, and mission controllers (Basner et al., 2015). The survey administered in campaign 2 (Analog Survey) was a survey of alertness and affect conducted using the REDCap survey tool hosted at the University of Pennsylvania (Harris et al., 2009). This survey contains 18 questions and is identical to the survey currently administered in our studies in Antarctic research stations (Concordia, Halley, Neumayer). Both surveys asked subjects to report their time in bed on the previous night; their caffeine, alcohol, or medication consumption; and asked them to rate their tiredness. In addition, the expanded survey in campaign 2 asked subjects to rate their perceived sleep quality, workload, sleepiness, happiness, sickness, physical exhaustion, mental fatigue, stress, depression, boredom, loneliness, monotony, and whether they had any conflicts with fellow crewmembers in the past week and how many of those were resolved. Supplementary Table 1 lists the different survey questions in full.

### Statistical Analysis

For each of the 10 Cognition tests, we selected one key accuracy outcome and one key speed outcome. The definitions of these variables can be found in Supplementary Table 2. Statistical analyses were performed with SAS (version 9.3, SAS Institute, Carey, NC, United States). If not noted otherwise, we ran mixed effects models of variance (Proc Mixed) with random subject effect. Model residuals were checked for normality via visual inspection of QQ-plots. In rare instances, residuals deviated relevantly from a normal distribution. In these cases, we decided to nevertheless perform the statistical analyses on untransformed data for two reasons: (1) Mixed models are very robust relative to violations of the assumption of normally distributed residuals. (2) Transformations fundamentally change the nature of the variable, making the interpretation of the results considerably more complex. If deemed necessary, statistical comparisons were adjusted for α-inflation due to multiple testing with the false discovery rate method (Curran-Everett, 2000).

#### Effects of Age, Sex, and Educational Attainment

To investigate the effects of age, sex, and education on key metrics of accuracy and speed in the Cognition test battery, we averaged test results for each outcome across the first seven days in mission within subjects. To facilitate data pooling across campaigns, testing sessions affected by sleep deprivation were excluded for campaign 1, and testing sessions performed on mission days 8–14 were excluded for campaign 2. The within-subject means were then subjected to a multivariable linear regression with the following independent variables:

• Age (continuous variable),
• Sex (categorical: male, female), and
• Educational attainment (categorical: Bachelor’s degree, Master’s degree, Ph.D. or equivalent).

#### Time in Mission Effects

To investigate the effects of time in mission on key metrics of accuracy and speed in the Cognition test battery, individual test scores were z-transformed (excluding the familiarization testing session) to facilitate comparisons across outcomes and tests. Sleep deprivation testing sessions were excluded from the analysis. We ran mixed effects models of variance with random subject effect with testing session number as an independent variable.

#### Sleep Deprivation Effects

To investigate the effects of sleep deprivation on key metrics of accuracy and speed in the Cognition test battery, we ran two separate models for campaigns 1 and 2 due to the different nature of the sleep challenge in the two campaigns (see Figure 1).

In campaign 1, sleep onset was delayed on mission day 3, and crewmembers only retired after they performed a Cognition battery session at 2:33 am on mission day 4 (administration time averaged across subjects). The following sleep period was shorter than those typically scheduled in HERA (see Figure 1). The sleep period between mission days 4 and 5 had a normal duration.

In campaign 2, subjects were sleep deprived for one full night, i.e., they did not retire on mission day 10, spent the next whole night and day awake, and only retired on the evening of mission day 11. The following sleep period was 2 h longer than usual.

For each campaign, the data set was restricted to the four test administrations outlined in Figure 1. We then ran a mixed effects model with random subject intercept with condition as a categorical independent variable and adjusting for subject age, sex, and educational attainment.

## Results

### Effects of Age, Sex, and Educational Attainment

The only statistically significant effect was found for age and cumulative reaction time on the VOLT. With increasing age, subjects took 0.42 s longer per life year to complete the VOLT (*p* = 0.0121). After adjusting for Type-I error inflation due to multiple testing with the false discovery rate method (*N* = 20 comparisons), this effect did not survive at *p* < 0.05 (Curran-Everett, 2000).

### Time in Mission Effects

**FIGURE 2:**
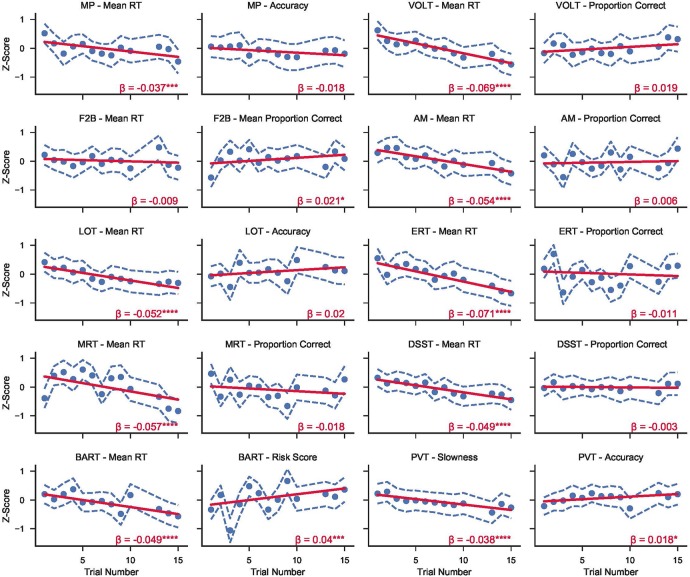
Z-transformed standard speed and accuracy metrics from the 10 tests in the Cognition battery over time in mission across both campaigns at HERA. Circles refer to the mean score across subjects from both campaigns for each trial number. Dashed blue lines show the 95% CI range around each mean point. Solid red lines represent mixed effects linear models fit to the data for each variable. *β* refers to the linear slope of the regression model (i.e., change in z-score per trial). Asterisks refer to significance levels after adjusting for multiple testing with the false discovery rate method with *p*-values referring to H_0_: regression line slope = 0. (*p < 0.05; **p < 0.01; ***p < 0.001; ****p < 0.0001).

The effects of time in mission on 10 key Cognition accuracy and 10 key speed outcomes are shown in Figure 2. Regression lines are based on a model with trial number as the only predictor (except for sleep deprivation days, one survey was performed each day of the mission). Sleep deprivation test sessions were excluded from the analysis. Subjects statistically significantly increased speed across test administrations on all tests but the F2B [operationalized as slowness (10 – mean reciprocal reaction time) for the PVT, or mean reaction time for all other tasks]. Test accuracy remained unchanged across administrations except for the PVT and the F2B. Accuracy on the F2B and the PVT both increased significantly over time in mission, albeit with small effect sizes (*β* = 0.021, and *β* = 0.018 respectively), and these effects survived adjustment for multiple testing. Finally, while BART risk score is listed among the accuracy variables, the significant increase across time in mission actually indicates that subjects took more risks on average on this task with time in mission (*β* = 0.04), and this effect did survive adjustment for multiple testing.

**FIGURE 3:**
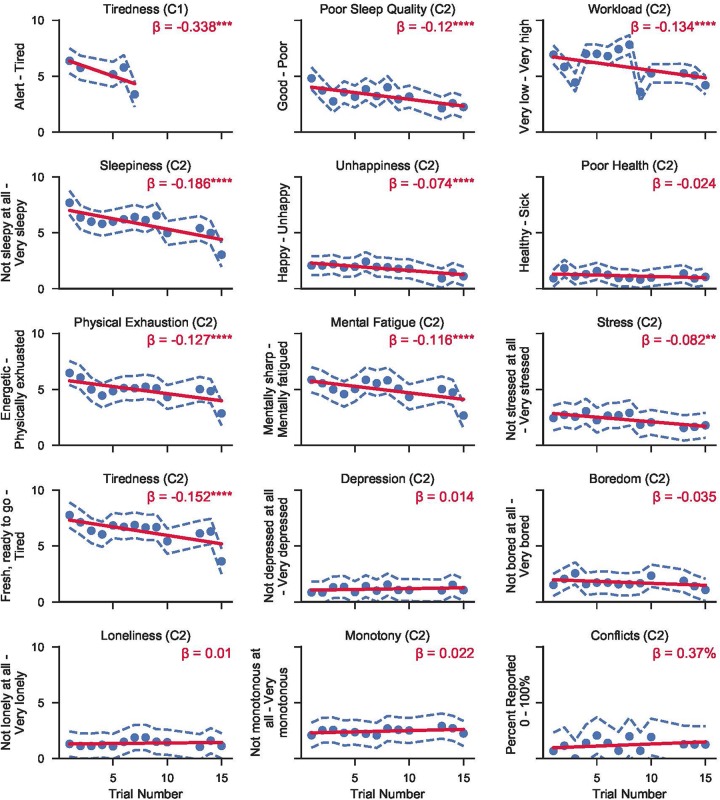
Raw (untransformed) survey response scores from the surveys administered in either campaign 1 (C1) or campaign 2 (C2) at HERA. Circles refer to the mean score across subjects from either campaign for each trial number. Dashed blue lines show the 95% CI range around each mean point. Solid red lines represent mixed effects linear models fit to the data within each question. *β* refers to the linear slope of the regression model (i.e., change in raw score per trial). Asterisks refer to significance levels after adjusting for multiple testing with the false discovery rate method with *p*-values referring to H_0_: regression line slope = 0. (*p < 0.05; **p < 0.01; ***p < 0.001; ****p < 0.0001).

Estimated marginal means across time in mission from the surveys administered prior to each Cognition battery are shown in Figure 3. Regression lines are based on a model with trial number as the only predictor (except for sleep deprivation days, one survey was performed each day of the mission). Sleep deprivation test sessions were excluded from the analysis.

The following survey outcomes showed a significant linear trend across survey administrations: decreasing ratings of tiredness for both campaigns; as well as decreasing ratings of sleepiness, physical exhaustion, mental fatigue, stress, workload, poor sleep quality, and unhappiness in campaign 2.

There were no significant trends for ratings of health, depression, boredom, loneliness, monotony, and conflicts across survey administrations. In general, crewmembers rated themselves happy, healthy, not stressed, not depressed, not bored, not lonely, with low levels of monotony, and high levels of sleep quality. Crew conflicts were reported on ≈1 out of 10 surveys. Ratings of tiredness, workload, sleepiness, physical exhaustion, and mental fatigue were medium to high, but decreased with time in mission.

### Sleep Deprivation Effects

**FIGURE 4:**
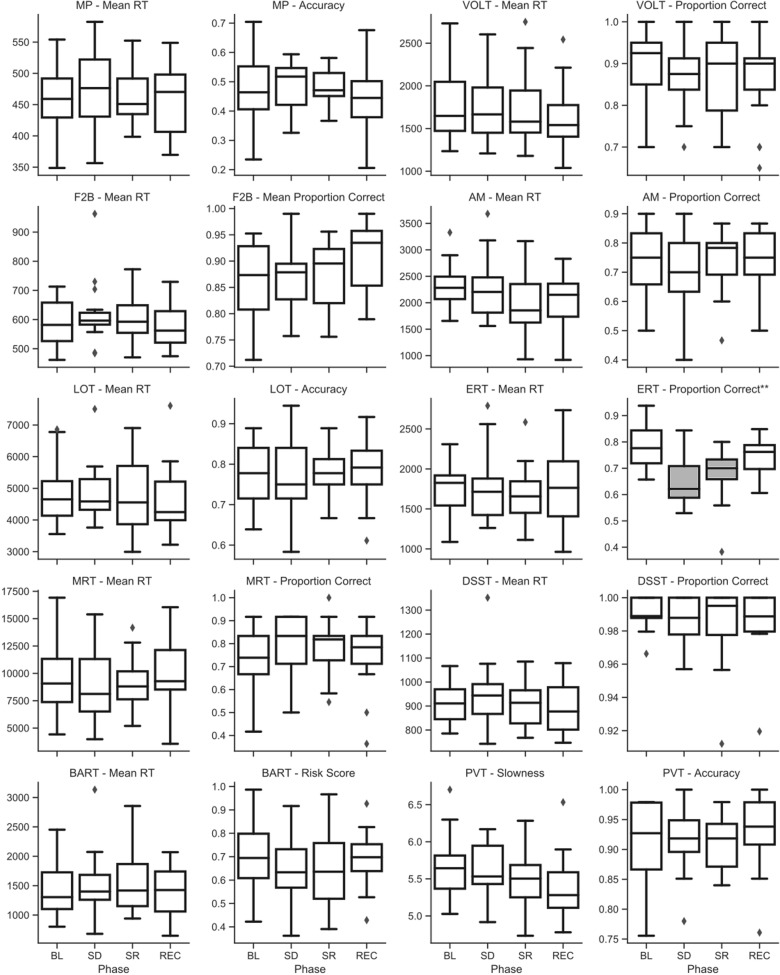
Raw (untransformed) scores across sleep deprivation phases in campaign 1 of HERA on key speed and accuracy metrics of the Cognition battery. Asterisks in the sub-plot title refer to type-III significance levels of sleep deprivation as a main effect after adjusting for multiple testing with the false discovery rate method (*p < 0.05; **p < 0.01; ***p < 0.001; ****p < 0.0001). In plots with a significant omnibus effect, data shaded in gray were significantly different from baseline (BL) in a *post hoc t*-test.

**FIGURE 5:**
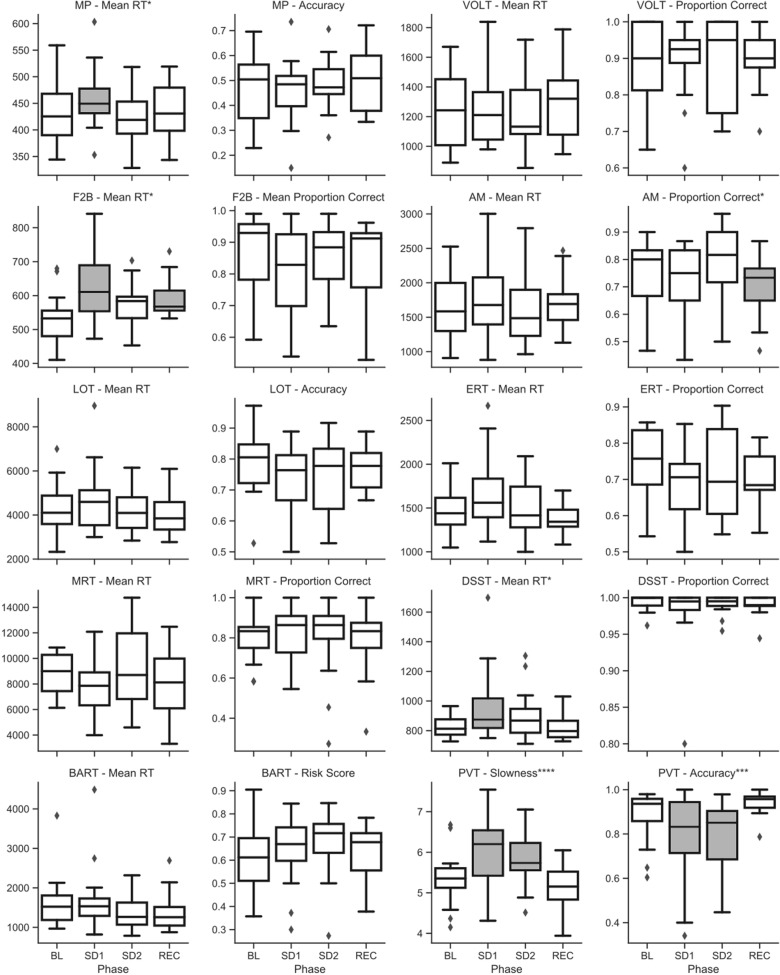
Raw (untransformed) scores across sleep deprivation phases in campaign 2 of HERA on key speed and accuracy metrics of the Cognition battery. Asterisks refer to type-III significance levels of sleep deprivation as a main effect after adjusting for multiple testing with the false discovery rate method (*p < 0.05; **p < 0.01; ***p < 0.001; ****p < 0.0001). In plots with a significant omnibus effect, data shaded in gray were significantly different from baseline (BL) in a *post hoc t*-test.

Raw (untransformed) scores across sleep deprivation phase for key metrics of speed and accuracy on the Cognition battery are shown in Figure 4 for campaign 1 and Figure 5 for campaign 2. *P*-values for all type-III fixed effects and *post hoc* tests are shown in Supplementary Table 3 for campaign 1 and in Supplementary Table 4 for campaign 2. Additionally, individual subject trends for key metrics of speed and accuracy on the battery are shown in Supplementary Figure 1 for campaign 1, and Supplementary Figure 2 for campaign 2.

In campaign 1, the ERT showed a significant effect of the sleep challenge, with significantly fewer emotions correctly identified after sleep deprivation and sleep restriction compared to both baseline and recovery (see Figure 4). This effect survived at *p* < 0.01 after adjusting for multiple testing. A closer look at the different emotion categories showed significant effects of sleep deprivation for displays of all emotion categories and neutral stimuli. However, these effects were not statistically significant (*p* > 0.05) for displays of happiness and sadness. No other standard speed or accuracy outcomes of the Cognition battery showed a significant main effect of the sleep challenge in campaign 1.

In campaign 2, there was a statistically significant and robust effect of sleep deprivation on the PVT in both speed and accuracy. These effects survived at *p* < 0.0001 and *p* < 0.001 after adjusting for multiple testing, respectively. Subjects were significantly slower and less accurate during both sleep deprivation sessions compared to baseline and recovery (see Figure 5).

On the DSST and the MP, subjects were significantly slower during sleep deprivation in campaign 2 (see Figure 5). This effect survived at *p* < 0.05 after adjusting for multiple testing. In both cases, *post hoc t*-tests indicated that subjects were significantly slower during the first sleep deprivation session compared to baseline, but that their speed recovered somewhat during the second sleep deprivation session the following evening.

Although type-III tests of fixed effects indicated statistically significant differences between conditions (some of them surviving adjustment for multiple testing) for the F2B and the AM, no clear pattern emerged from *post hoc* analyses with consistently higher performance during baseline and recovery and lower performance during sleep deprivation and restriction (see Figure 5).

**FIGURE 6:**
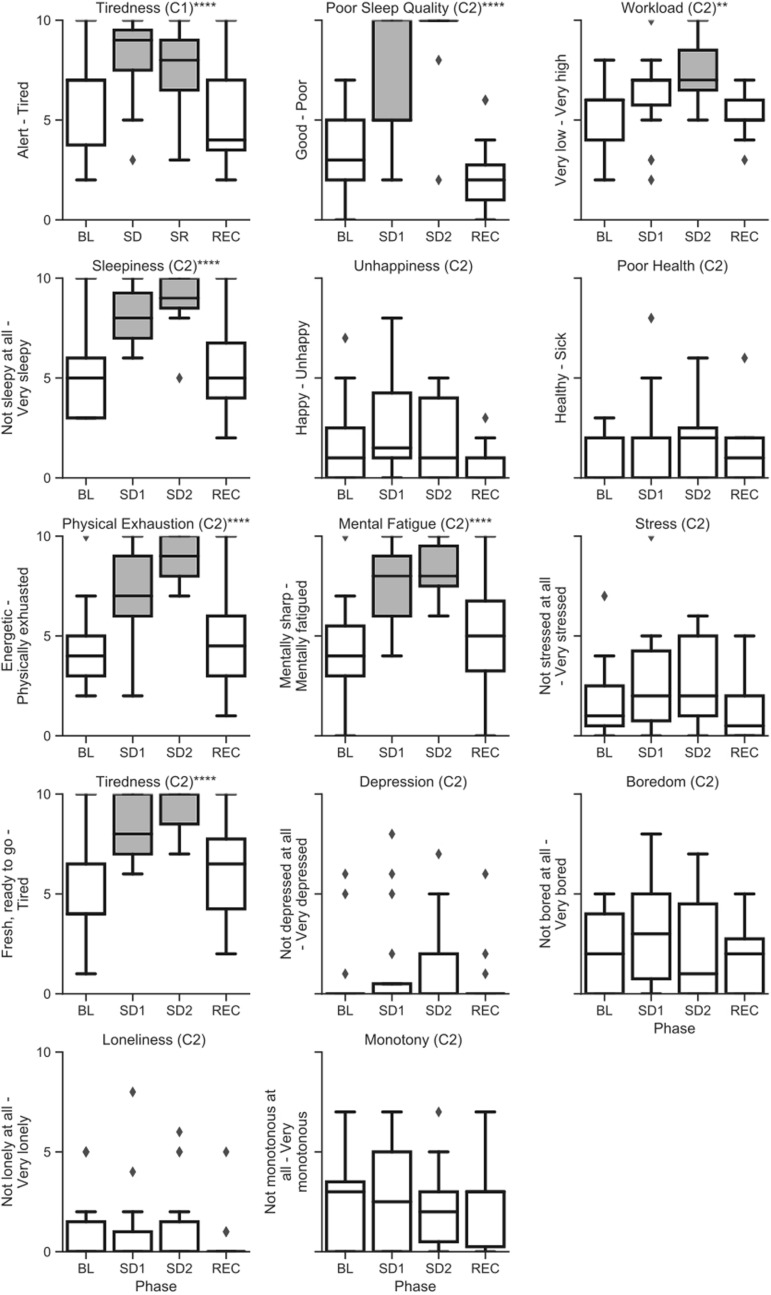
Raw (untransformed) scores from the alertness and affect surveys across sleep deprivation phases in campaign 1 (C1) and campaign 2 (C2) of HERA. Asterisks refer to type-III significance levels of sleep deprivation as a main effect after adjusting for multiple testing with the false discovery rate (*p < 0.05; **p < 0.01; ***p < 0.001; ****p < 0.0001). In plots with a significant omnibus effect, data shaded in gray were significantly different from baseline (BL) in a *post hoc t*-test.

Raw (untransformed) scores across sleep deprivation phase for alertness and mood survey questions are shown in Figure 6. Additionally, individual subject trends for subjective affect on the survey are shown in Supplementary Figure 3. Subjects were significantly more likely to report tiredness during the sleep challenge in both campaigns, and the longer survey in campaign 2 also showed a significant effect of sleep deprivation on subjects reporting poor sleep quality, increased workload, sleepiness, physical exhaustion, and mental fatigue. Conversely, there was no significant effect of the sleep deprivation on subjects reporting unhappiness, poor health, stress, depression, boredom, loneliness, monotony, or the prevalence of crew conflicts. *P*-values for all type-III and *post hoc* tests for the surveys are reported in Supplementary Table 5.

## Discussion

### Effects of Age, Sex, and Educational Attainment

The only statistically significant effect of age, sex, and educational attainment on key Cognition speed and accuracy test outcomes was seen for the VOLT, where the time needed for subjects to complete the test increased with age. The fact that we did not find any other statistically significant effects related to age, sex, or education is likely based on the low sample size and the associated statistical power. *N* = 32 subjects is low for between-subject comparisons. The statistical model contained 5 variables, which, as a rule of thumb, requires at least *N* = 50 subjects to achieve statistically reliable results. Hence, it is not surprising that none of the subjective outcomes showed a statistically significant relationship with age, sex, or educational attainment. It is likely that we were underpowered to detect some of the effects that were described for the Cognition tests in the literature (Basner et al., 2015, 2018; Moore et al., 2017; Lee et al., 2020).

### Time in Mission Effects

With increasing time in mission, speed on all Cognition tests increased while accuracy remained unchanged or improved. Subjects were therefore getting faster on the tasks, rather than simply shifting their response strategy to prefer speed over accuracy. There was only one exception to this pattern: Speed on the F2B did not increase with time in mission. However, responses to stimuli on the F2B are restricted to a 1,750 ms response window, which can obscure some of the practice effects observed with the other Cognition tests. Additionally, the F2B and the PVT both showed modest but statistically significant improvement in accuracy over time in mission. In a normative study performed in astronauts, astronaut candidates, and mission controllers at JSC we did see increases in accuracy across test administrations for several of the other tasks in Cognition, which we did not find here (Basner et al., 2015). The increase in speed across administrations therefore reflects an expected practice effect from crewmembers who are coping with the mission scenarios, but it is unclear whether the lack of a concurrent improvement in accuracy on 8 of the 10 tests resulted from the mission stressors. Alternatively, as the normative studies reported by Basner et al. (2015) used a laptop platform, it is also possible that practice effects in the accuracy domain are less pronounced on a touch-screen platform.

It is typically possible to correct for expected practice effects on the Cognition battery given sufficient normative data. Such correction would be problematic in this study, however, given that the majority of normative data we’ve collected so far have been on the laptop platform. As mentioned previously, Moore et al. (2017) found some differences between the laptop and iPad iterations of the battery, especially on overall speed in the battery. While these differences do not impact comparisons within subjects on a single platform, they do render direct comparisons between the laptop and iPad platforms problematic.

Conversely, The PVT is known for having minimal practice and aptitude effects (Basner et al., 2018), but we found a significant improvement in both speed and accuracy over time in mission. As HERA is a simulated operational environment, it is unclear to what extent these practice effects can be explained by the specifics of the mission simulation. For example, sleep time increased slightly with time in mission during campaign 1 (McGuire et al., 2017), and feelings of tiredness and sleepiness decreased at the same time. The observed increase in response speed and decrease in the number of lapses were therefore more likely caused by increases in sleep duration and efficiency across the mission, which are also reflected in decreasing levels of tiredness, sleepiness, mental fatigue, and physical exhaustion with time in mission (see Figure 3).

Subjective ratings were generally positive across time in mission omitting the sleep deprivation sessions. Crewmembers rated themselves happy, healthy, not stressed, not depressed, not bored, not lonely, with low levels of monotony and high levels of sleep quality. Ratings of tiredness, workload, sleepiness, physical exhaustion, and mental fatigue were medium to high, but decreased with time in mission. Overall, the observed pattern suggests that, on average, crewmembers coped well with or adjusted well to the isolated and confined HERA environment with increasing time in mission, which is also corroborated by their stable performance on the Cognition battery in absence of the sleep challenge.

### Sleep Deprivation Effects

We found significant sleep deprivation effects on the ability to correctly identify emotions on the ERT, but only in campaign 1. Conversely, PVT response speed and accuracy, MP speed, and DSST speed decreased significantly during sleep deprivation, but only in campaign 2. We also found significant differences between sleep deprivation conditions for the F2B and AM in campaign 2, but these effects were less consistent. The stark differences in performance outcomes of the sleep challenge between campaigns is likely attributable to changes in the sleep challenge between campaigns. The sleep deprivation session in campaign 1 was collected roughly 3 h earlier at night than in campaign 2, and the session was immediately followed by a short sleep opportunity compared to total sleep deprivation in campaign 2. Consequently, both the difference in circadian phase and the move from sleep restriction to total sleep deprivation between campaigns likely caused this discrepancy.

The PVT and DSST were already identified as the two tests with the highest sensitivity to sleep loss, both in general (Lim and Dinges, 2010) as well as on the Cognition test battery specifically (Basner et al., 2015), so the effect of total sleep deprivation on these tasks in campaign 2 is not surprising, while the sleep challenge in campaign 1 may not have been severe enough to elicit the same effect.

Conversely, the ERT was the only test to show significant performance decrements in campaign 1. We did not see this effect on the ERT in a sample of 44 subjects undergoing one night of total sleep deprivation (Basner et al., 2015). However, in that study, subjects performed Cognition shortly after 11 am when the circadian system had started to promote alertness. The fact that sleep deprivation can affect emotion recognition has been reported in the literature (van der Helm et al., 2010; Cote et al., 2014). While this effect was not detected in the harsher sleep deprivation challenge of campaign 2, the larger degree of variability in performance on the ERT in campaign 2 may have masked an individually variable effect (which can be seen in a subset of subjects as demonstrated in Supplementary Figure 2), but the current data were inconclusive on this point. It is notable, however, that such an effect would be rather valuable to detect as a risk factor for crew cohesion on autonomous long-duration exploration class missions, especially given that this cognitive domain is historically under-examined in the spaceflight cognitive performance literature.

It is also worth noting that the sleep challenge in HERA was integrated into the scenario of the mission simulation itself, and subjects were given a mission-relevant reason for extending work hours, as well as tasks that occupied at least part of that time. The effect of the sleep challenge itself is therefore inherently tied to the overall mission effect in this study, as well as to the effects of the concurrent confinement and relative isolation. On the one hand, this experimental design complicates direct comparisons to sleep deprivation effects in other studies. Importantly, however, this design also affords greater ecological validity to the context in which sleep challenges actually occur in spaceflight (e.g., as a result of preparing for extra vehicular activities or docking maneuvers).

Finally, we also found significant sleep deprivation effects for subjective ratings of tiredness in both campaigns, and for sleepiness, physical exhaustion, mental fatigue, sleep quality, and workload in campaign 2. Ratings of happiness, health, stress, depression, boredom, loneliness, and monotony in campaign 2 did not differ significantly with sleep deprivation phase. Taken together, these data suggest that subjects in campaign 2 found their experience of their physical and mental fatigue particularly salient during their sleep deprivation, but that they did not seem to note a spillover of this fatigue into their affect and health more broadly.

Although we typically ask subjects to report the quality of their *last* sleep period, the reported decrease in sleep quality may be simply related to the fact that subjects did not sleep at all between survey administrations during the sleep deprivation period, which they interpreted as “poor sleep quality”. Alternatively, assessments of the same prior sleep period may have deteriorated both due to sleep deprivation and/or due to the increasing time period between the sleep period itself and the time of the survey.

## Conclusion

The current findings suggest that the stressors of confinement and relative isolation of up to two weeks with a work schedule comparable to that on the ISS, as administered in HERA, may not show a significant negative impact on cognitive performance in any of the domains examined by Cognition. However, it is not possible at this point to determine whether there was no mission effect or whether practice effects masked a true mission effect. The fact that practice effects in the accuracy domain were only found for two out of the 10 Cognition tests (in contrast to previous findings of a pronounced practice effect on several of the other tasks) may suggest there was a mission effect. On the other hand, the use of a touch screen based test platform could also at least partly explain this discrepancy. Efforts to generate data for correcting scores on the Cognition test battery for practice and stimulus set difficulty effects are currently underway.

Given that a typical ISS mission has often been ≈6 months in duration, and that longer duration exploration class missions would be expected to last well in excess of a year, further work is necessary to investigate whether this negative finding holds true for longer missions, but the current data establish a minimum threshold for detecting significant effects of time in mission with the Cognition test battery.

Conversely, a single night of total sleep deprivation was shown to have a significant impact on psychomotor vigilance (PVT) and cognitive throughput (DSST) in an astronaut-like population both in the early morning after sleep deprivation and at the end of the following day. These findings suggest that special care is warranted to ensure adequate sleep for crew members especially around mission-critical events like dockings and extra-vehicular activities. There may be a sleep deprivation threshold in between the durations administered in campaigns 1 and 2 in this study that would impair vigilance and cognitive speed in the crew members.

Taken together, the findings from this study underscore the importance of using cognitive testing tools designed specifically for the astronaut population, and with a breadth of cognitive domains surveyed. Different stressors in the spaceflight environment may impact various cognitive domains differently, and many generic cognitive tests may not have the sensitivity or the skill ceiling to detect these effects in the highly skilled and highly motivated astronaut population.

## Data Availability Statement

The raw data supporting the conclusions of this manuscript can be requested by qualified researchers from NASA’s Life Science Data Archive (https://lsda.jsc.nasa.gov/).

## Ethics Statement

The studies involving human participants were reviewed and approved by the NASA Institutional Review Board. The patients/participants provided their written informed consent to participate in this study.

## Author Contributions

MB, DD, and RG contributed to the study design. JN, MB, and EH contributed to the data collection. JN, MB, and EH contributed to the database management. MB and TM contributed to the data analysis. JN, MB, EH, TM, and RG contributed to the results interpretation. JN and MB contributed to the manuscript draft. JN contributed to the figures. EH, DD, TM, and RG contributed to the manuscript revisions.

## Conflict of Interest

The authors declare that the research was conducted in the absence of any commercial or financial relationships that could be construed as a potential conflict of interest.
