# Supplementary figures and images for: Cognitive Performance During Confinement and Sleep Restriction in NASA’s Human Exploration Research Analog (HERA)
Source: Front Physiol. 2020 Apr 28;11:394. doi: 10.3389/fphys.2020.00394 (PMC7198903; doi:10.3389/fphys.2020.00394)

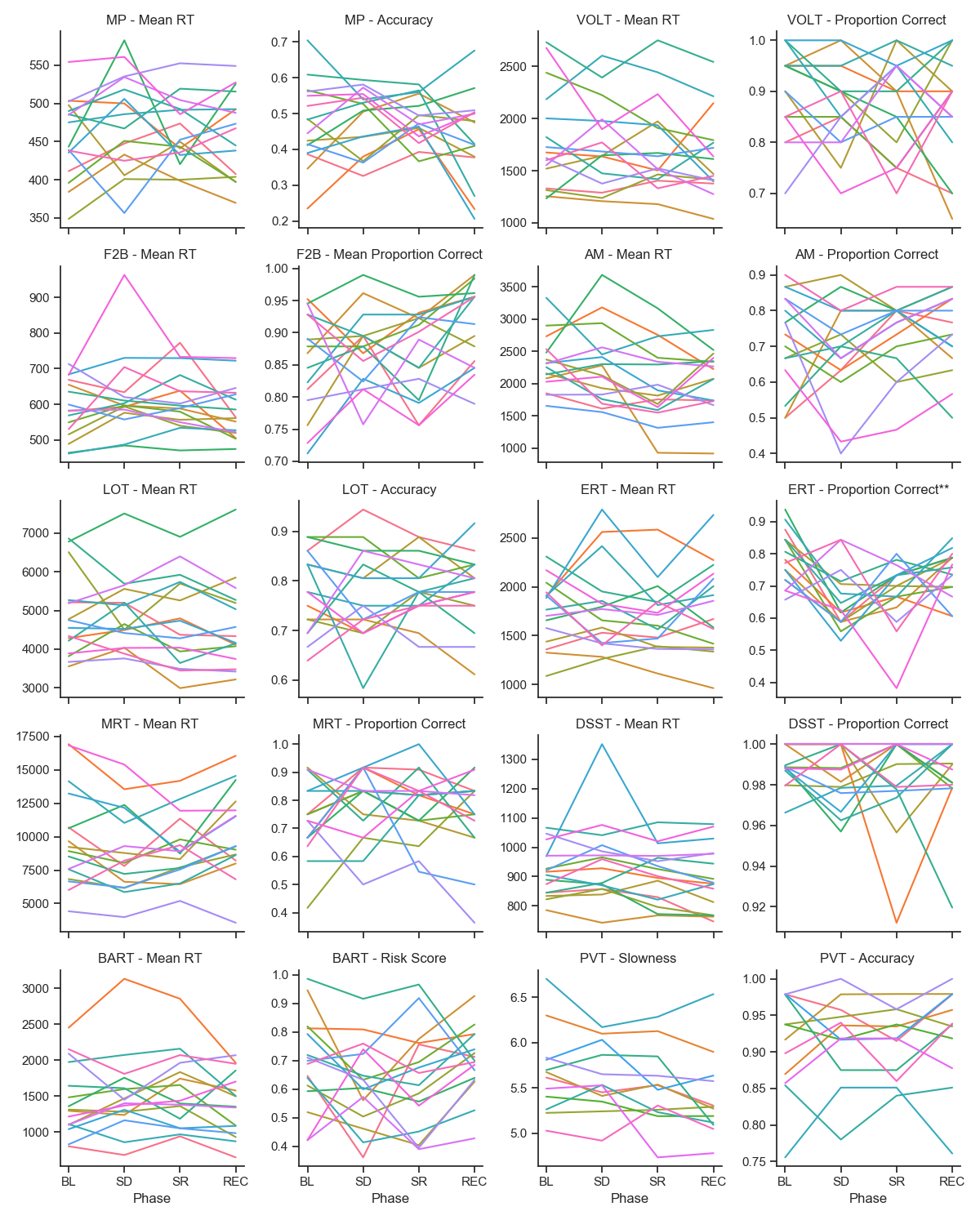

Supplement: Supplementary file 6 [file Image_1.jpeg]

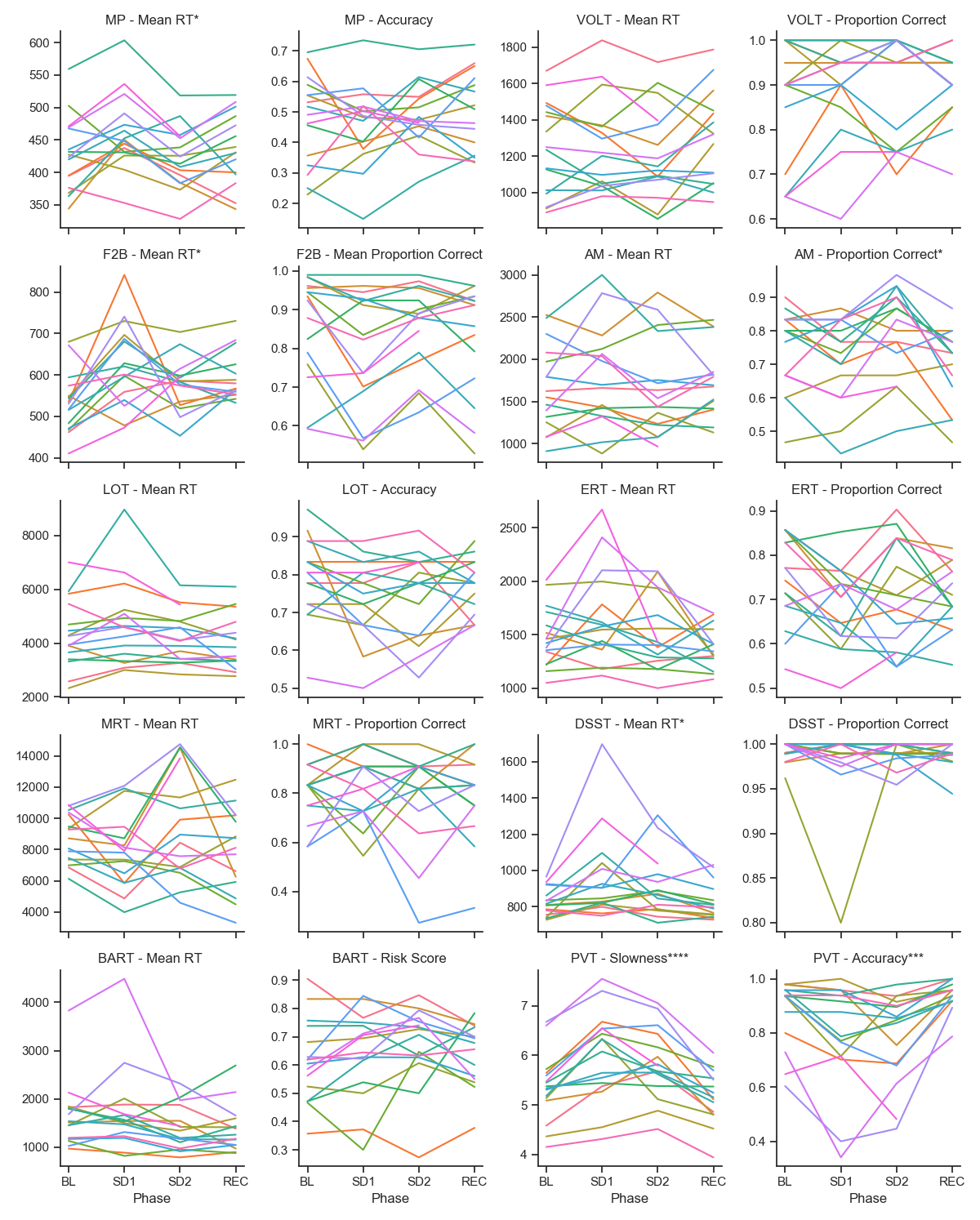

Supplement: Supplementary file 7 [file Image_2.jpeg]

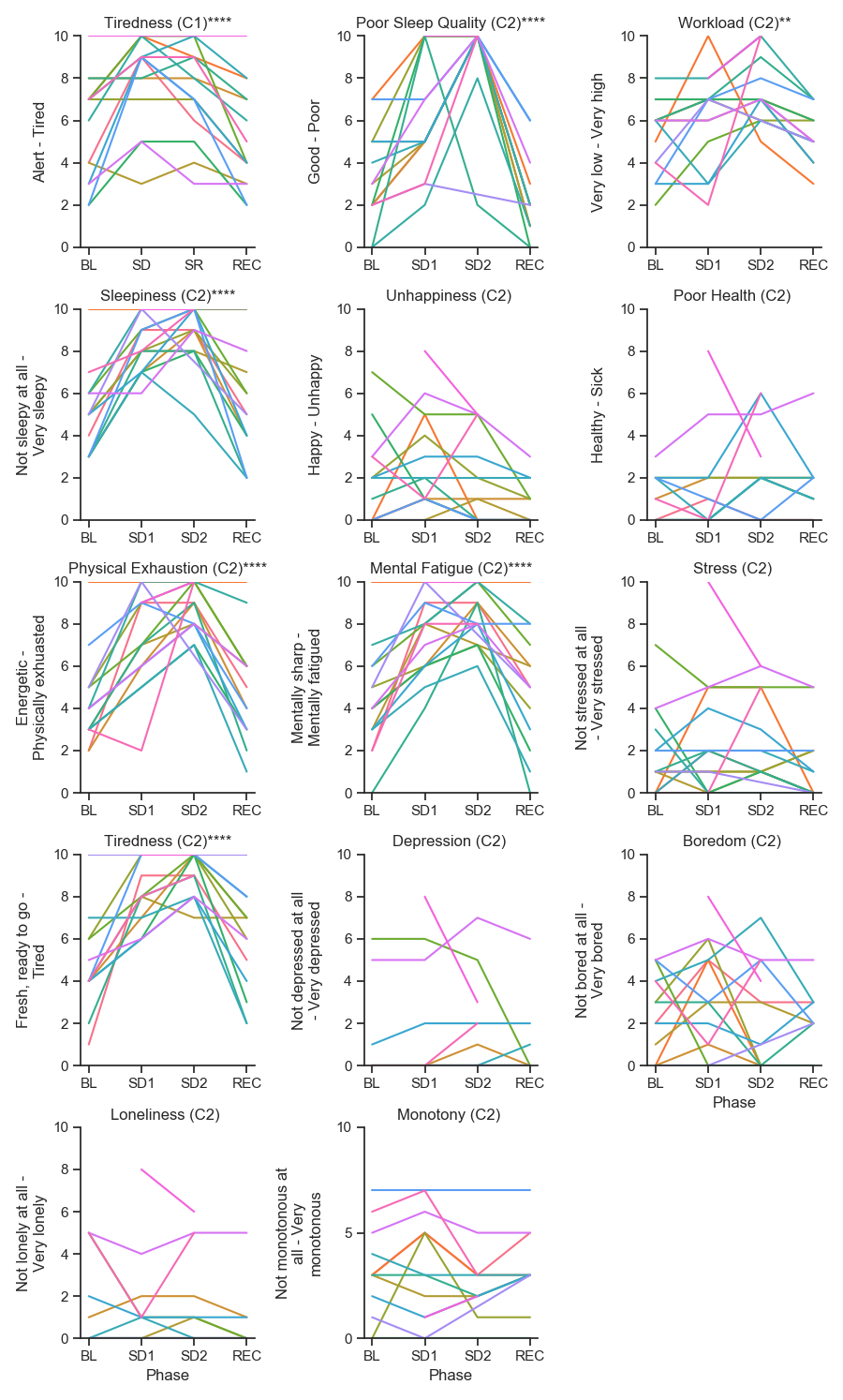

Supplement: Supplementary file 8 [file Image_3.jpeg]
